# Linking oscillations in cerebellar circuits

**DOI:** 10.3389/fncir.2013.00125

**Published:** 2013-07-29

**Authors:** Richard Courtemanche, Jennifer C. Robinson, Daniel I. Aponte

**Affiliations:** Department of Exercise Science, Groupe de Recherche en Neurobiologie Comportementale/Center for Studies in Behavioral Neurobiology, Concordia UniversityMontréal, QC, Canada

**Keywords:** oscillations, cerebellum, synchronization, sensorimotor interactions, network activity

## Abstract

In many neuroscience fields, the study of local and global rhythmicity has been receiving increasing attention. These network influences could directly impact on how neuronal groups interact together, organizing for different contexts. The cerebellar cortex harbors a variety of such local circuit rhythms, from the rhythms in the cerebellar cortex *per se*, or those dictated from important afferents. We present here certain cerebellar oscillatory phenomena that have been recorded in rodents and primates. Those take place in a range of frequencies: from the more known oscillations in the 4–25 Hz band, such as the olivocerebellar oscillatory activity and the granule cell layer oscillations, to the more recently reported slow (<1 Hz oscillations), and the fast (>150 Hz) activity in the Purkinje cell layer. Many of these oscillations appear spontaneously in the circuits, and are modulated by behavioral imperatives. We review here how those oscillations are recorded, some of their modulatory mechanisms, and also identify some of the cerebellar nodes where they could interact. A particular emphasis has been placed on how these oscillations could be modulated by movement and certain neuropathological manifestations. Many of those oscillations could have a definite impact on the way information is processed in the cerebellum and how it interacts with other structures in a variety of contexts.

## INTRODUCTION

Oscillations are an important influence shaping local circuits in the brain (Buzsaki and Draguhn, 2004; Buzsaki, 2006). In recent years, various oscillatory phenomena have been identified as influential pattern synchronizers in the spinal cord, in the cerebral cortex, in the basal ganglia, and the cerebellum. Here, we will describe the various constitutive oscillatory phenomena in the cerebellar cortex, the main interactions that could take place in the cerebellar cortex between them, attempt to predict the resulting effects at the cerebellar output in the context of sensorimotor behavior, and then propose how oscillations in the cerebellum could contribute to pattern synchronizing across sensorimotor and cognitive systems.

The basic questions on neural coding that are current, in areas such as the cerebral cortex circuits, the hippocampal and parahippocampal structures, the olfactory system, and the amygdala (e.g., Collins et al., 2001; Perez-Orive et al., 2002; Harris et al., 2003; Glasgow and Chapman, 2007; Goutagny et al., 2009), are also key for the cerebellum. How do cerebellar cortex neurons shape into a population to form one of its many coherent representations at a given moment in time? What is the time-specific signature of cerebellar populations? Strong hints have been offered by the study of olivocerebellar interactions, showing that these ultimately produce intricate spatiotemporal patterns in Purkinje cell (PC) population coding to serve the task at hand (Welsh et al., 1995; Welsh, 2002). Considering the massively parallel modularity of the cerebellar cortex, we raise the question of how this complementarity could contribute to population coding via the various afferent systems and local circuit interactions. As has been demonstrated for olivocerebellar interactions, other existing oscillations are likely going to play a role in shaping cerebellar cortex population patterns. In addition, coherent long-range communication mechanisms are advantageous for a large structure such as the cerebellum, in order to coordinate its internal activity with other oscillatory sensorimotor networks. The temporal modulation of cerebellar population activity will certainly come into play in the capacity of the cerebellum to participate in sensorimotor transformations.

## DIFFERENT TYPES OF OSCILLATIONS IN THE CEREBELLAR CORTEX

When studying oscillations in cerebellar circuits, a significant discovery was that harmaline administration produced hyperrhythmic olivocerebellar activity (De Montigny and Lamarre, 1973; Llinás and Volkind, 1973). This line of inquiry has led to a systematic exploration of population coding in olivocerebellar circuits (for an example of a recent review, see Llinás, 2009). In contrast with the study of olivocerebellar interactions, for a long time there was a silent echo to such oscillatory phenomena in the other components of the cerebellar circuitry. This became particularly more apparent considering the interest in peri-movement cerebral cortex oscillations (Sanes and Donoghue, 1993). These oscillations were not mirrored by similar comparable phenomena in the cerebellum: this had been noted by at least one voice (Bullock, 1997). The finding of granule cell layer (GCL)-specific oscillations (Pellerin and Lamarre, 1997; Hartmann and Bower, 1998) rekindled an interest in the diversity of the oscillatory phenomena in the cerebellum. However, as reported in the historical perspective of Isope et al. (2002), certain cerebellar oscillations had actually been discovered long ago. In this paper, with the recent reemergence of multiple oscillation patterns in the cerebellar cortex circuitry (de Zeeuw et al., 2008; D’Angelo et al., 2009), we review the potential influences that these mechanisms and their interactions could have in the formation of cerebellar patterns of activity.

**FIGURE 1:**
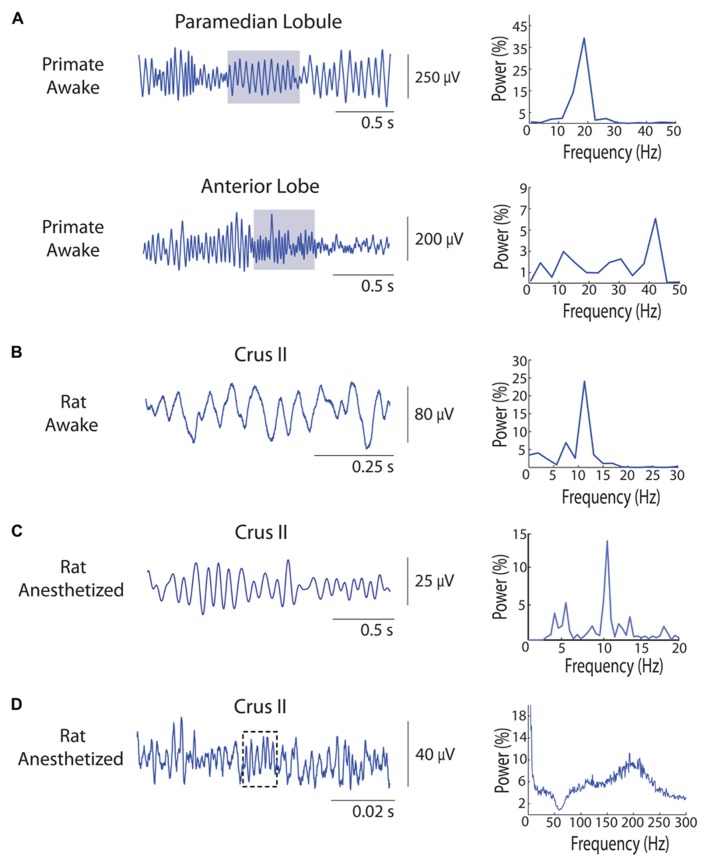
**A sample of oscillations recorded from cerebellar cortex *in vivo* local field potentials (LFPs) using metal microelectrodes.** LFP example data located on the left, and corresponding Fast Fourier Transform (FFT) spectrum on the right. FFT shown in the form of %. **(A)** Simultaneous different types of LFP oscillations in the primate rhesus monkey cerebellum. Top: LFP oscillations from the PM GCL, around 19 Hz. Bottom: faster LFP oscillations recorded in the anterior lobe GCL, going up to 40 Hz. Gray shaded area corresponds to the time period for the FFT analysis. Notice the simultaneous co-existence of two different oscillatory phenomena. **(B)** Recording of LFP oscillations in the awake rat cerebellar cortex GCL. In this sample, the signal oscillates around 10.5 Hz, FFT on the whole trace. **(C)** Recording of LFP oscillations in the urethane-anesthetized rat cerebellar cortex GCL. Oscillations are here around the same frequency, at 11 Hz, FFT on the whole trace. **(D)** Recording of fast LFP oscillations in the urethane-anesthetized rat cerebellar cortex, using differential metal microelectrodes separated by 500 μm, with at least one tip located approximately in the Purkinje cell layer. A 312 Hz short 6-cycle episode is highlighted. FFT averaged on 120 2-s windows, so for the whole 2 min.

**FIGURE 2:**
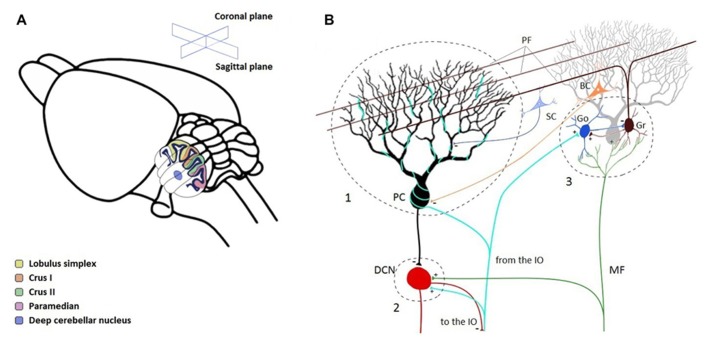
**Description of the cerebellar cortex substrate for local field potential oscillation interactions.** **(A)** General cerebellar organization, here from a rodent brain. Potent LFP GCL oscillatory sites can be recorded from within the cerebellar cortex posterior lobe, including the crus lobules, Crus II and Crus I, and the paramedian lobule (PM). For both the primate and the rodent, the PM lobule is a common GCL LFP oscillation recording site, at the level where the virtual slice is taken. **(B)** Cerebellar circuitry, with an emphasis on the potential interaction sites between different oscillatory phenomena. Highlighted are the Purkinje cell (PC) afferent region (1), the deep cerebellar nuclei (2, DCN), and the Golgi cell (Go)–granule cell (Gr) circuits in the GCL (3). Interactions are discussed in the text. IO, inferior olive; MF, mossy fiber; PF, parallel fiber; SC, stellate cell; BC, basket cell, the latter two provided for context.

A quick graphical illustration of the oscillatory phenomena can be presented here, admittedly by staying in the context of our recordings, with mainly the (1) granule cell layer 4–25 Hz oscillations in the primate paramedian lobule – PM, (2) the similar oscillation patterns at 4–25 Hz in the rodent, and (3) the Purkinje-cell layer fast (~150–300 Hz) oscillations being considered. **Figure 1** illustrates the local field potential (LFP) oscillations that can be recorded in the cerebellar cortex, here all recorded in the rhesus primate or laboratory rat *in vivo*. In the figure, it is apparent that the oscillatory phenomena in the primate cerebellar cortex GCL has a wide frequency band: already established in the range of 15–25 Hz, and recorded in the PM (see **Figure 2**), we also show that in the anterior lobe, certain sites show simultaneous oscillations at a higher frequency, in this case up to around 40 Hz (**Figure 1A**). This variety has not been much explored, and will warrant further investigation. The rodent version of these GCL oscillations has also been described, in the awake animal (~5–12 Hz, Figure 1B), but has also been characterized around the same frequencies under urethane anesthesia (**Figure 1C**). Finally, fast oscillations around 200 Hz, a more recent phenomenon, have been described for the PC layer (PCL), here also recorded under anesthesia (see **Figure 1D**). A representation of the involved structures and circuitry involved in those recordings is shown in **Figure 2**.

A large component of the cerebellar literature concerning oscillations between 4 and 30 Hz has been characterized by the subthreshold oscillatory activity in the inferior olive (IO), with near 10 Hz frequencies. As these have been well studied, *in vivo* and *in vitro*, only a broad characterization will be given here, having been well reviewed by the authors [e.g., in motor control (Llinás et al., 1991; Welsh and Llinas, 1997; Llinás, 2009), and also in mechanistic terms (Llinás et al., 1981; Llinás and Sugimori, 1992; Jacobson et al., 2008)]. Additionally, in the past few years, additional rhythmic phenomena have appeared *in vivo* in the cerebellar cortex GCL, which have subsequently been investigated *in vitro*. We will describe some main points of olivocerebellar activity first, then the GCL oscillation at similar frequencies. Finally, in addition to these firmly established oscillatory phenomena, we will address the fast oscillations in the PCL (e.g., de Solages et al., 2008), the ultra-slow fluctuations in electrophysiological activity (Chen et al., 2009), and the slow cerebro-cerebellar membrane potentials (Ros et al., 2009). We will describe each of those oscillatory phenomena in quasi-chronological succession of their first report.

### OLIVOCEREBELLAR RHYTHMICITY

The IO, located in the ventral brainstem, has long been studied for its powerful connection via climbing fibers (CFs) to contralateral PCs in the cerebellum. It is one of the strongest synaptic connections in the central nervous system (Llinás, 2009); an IO neuron may synapse with up to 10 PCs (Armstrong and Schild, 1970), but each PC only has one CF which intimately connects to its soma and dendritic arbor (see **Figure 2**). CF activation of PCs generates atypical action potentials, known as complex spikes (CS), that are characterized by having large amplitudes and subsequent wavelets. The timing of CF activation, under normal conditions firing at 1 Hz, is considered to be an important variable in determining cerebellar cortex information coding.

The IO has important intrinsic rhythm capabilities. Some of the first studies indirectly observed the IO as an oscillator using harmaline as a means to enhance the IO rhythmicity (Lamarre et al., 1971; De Montigny and Lamarre, 1973). Under normal conditions, the IO nucleus oscillates at a subthreshold 10 Hz (Devor and Yarom, 2002; Chorev et al., 2007). Animals receiving systemic harmaline, a psychoactive alkaloid, produced rhythmic CSs at ~10 Hz, coming from CFs (Lamarre et al., 1971; De Montigny and Lamarre, 1973; Llinás and Volkind, 1973). These studies marked the beginning of a series of inquiries on the rhythmic properties of the olivocerebellar system. Typical spontaneous CS discharge at ~1 Hz, but harmaline transforms the subthreshold oscillations into coherent and sustained firing at ~10 Hz. This strong olivocerebellar rhythmic activation expresses itself as a systemic tremor of the animal [for a video of the phenomena, refer to Movie S1 from Park et al. (2010)]. Specifically, it was recently discovered that Ca_V_3.1 T-type Ca^2+^ channels may be the molecular substrate allowing for the IO to oscillate. Park et al. (2010) effectively showed that mice lacking the Ca_V_3.1 gene were not affected by systemic harmaline injections. This was confirmed electrophysiologically by recording the IO and deep cerebellar nuclei (DCN) *in vitro* in both wild-type and Ca_V_3.1^-/-^ mice. The cells of the IO have long been known to be electrically coupled together via gap junctions (Llinás et al., 1974), and therefore postulated to oscillate in clusters of neurons. Using voltage sensitive-dye technique to image populations of neurons, Leznik et al. (2002) demonstrated that clusters of IO neurons do oscillate in unison, at an average frequency of 1–7 Hz. Furthermore, external stimulation of the cell clusters consistently triggered an oscillatory reset; rather than change the frequency of oscillation, this external stimulation produced a phase shift in the subthreshold oscillations (Leznik et al., 2002). This could in turn synchronize CF activation of PCs in the cerebellum. The role of gap junctions in the IO is pivotal for the capacity to form clusters: blocking those gap junctions produces a disconnection of the IO clusters and virtually abolishes population oscillations without affecting the subthreshold oscillations of single cells (Leznik and Llinas, 2005).

In the same manner as thalamic and brainstem nuclei influence cortical systems via temporal patterns, it is interesting that a nucleus such as the IO can temporally influence a large neural sheet, such as the cerebellar cortex. IO rhythmicity could definitely contribute to the organization of network activity in the cerebellar cortex (Jacobson et al., 2008; Llinás, 2009). Recent models suggest that this network may be capable of influencing PCs at a much finer temporal resolution than 10 Hz (Jacobson et al., 2008, 2009). Their model posits that GABAergic input from the DCN, which decouples IO cells by acting on gap junctions (Lang et al., 1996), would set cells out of phase from each other. Since IO cells preferentially fire and optimize their influence on the PCs at the peaks of their subthreshold oscillations (Mathy et al., 2009), out of phase cells reaching threshold would do so staggered in time, greatly increasing their temporal resolution and influence on PCs. This could support the temporal detail needed for the timing of motor events (for full explanation, see Jacobson et al., 2008).

#### Functional roles of olivocerebellar oscillations

To evaluate the effects of these oscillations on cerebellar processing, a major technological advance was the creation of methods permitting the recording of CSs in arrays of PCs (Sasaki et al., 1989; Welsh et al., 1999), yielding population data in the awake behaving animal (Welsh et al., 1995). Recording CS activity in PCs provides an indirect confirmation of IO activity. This does not, however, inform on the nature of the organization of the IO network activity. Welsh et al. (1995) were able to correlate CS activity from matrices of PCs to animal behavior. Thus, the output of the IO system could be studied at a PC population level with regard to movement, informing on the effects of those connections to organize coherent cellular PC networks (Lang et al., 1996; Lang, 2002; Blenkinsop and Lang, 2006). In the awake animal, these olivocerebellar networks are organized under the influence of oscillations, namely in their parasagittal heightened synchrony (Lang et al., 1999). In the context of movement, these form organized networks, shaped as task-specific mosaics driven by oscillatory activity in the olivocerebellar system (Welsh et al., 1995).

Links have been made between this oscillatory activity and the IO working as a motor clock in health and disease (Welsh et al., 2005). However, there was resistance to the idea of the rhythmic activity of the IO working as a master motor clock (Keating and Thach, 1995, 1997). Recent additional evidence for the motor clock hypothesis came from tasks performed during brain imaging, where the IO functional magnetic resonance imaging (fMRI) activation can be related to the timing component of the tasks (Xu et al., 2006; Wu et al., 2011). There is also a role for the oscillations in olivocerebellar activity to modulate movement generation in the primary motor cortex (Lang et al., 2006b). However, while the timing of CS activity can be timed with movement parameters in the monkey (Kitazawa et al., 1998), in the context of rodent licking movements, certain CSs are not coherent with movement initiation in rhythmic licking (Bryant et al., 2010), somewhat different from Welsh et al. (1995). While the strict idea of a central clock is indeed difficult to prove or disprove, temporally constrained activity, more particularly rhythmic activity, should play an important role in timed sensorimotor or cognitive behavior (Steriade et al., 1993; Llinás, 2001; Paulsen and Sejnowski, 2006; Sejnowski and Paulsen, 2006).

Anatomically, olivocerebellar axons show a parasagittal plane orientation in their distribution (Oberdick et al., 1998; Apps and Garwicz, 2005), a motif matched by the patterns of specific protein expression such as zebrin (Hawkes and Leclerc, 1987; Leclerc et al., 1990; Hawkes et al., 1997). These anatomical patterns influence the flow of information across the cerebellar cortex (Apps and Hawkes, 2009; Ebner et al., 2012), and confer a sagittal modularity to the olivocerebellar activity, both confirmed in anesthetized and awake animals (Sasaki et al., 1989; Sugihara et al., 1995; Lang et al., 1999; Fukuda et al., 2001). This stripe of activity is spatiotemporally defined by the temporal exactness at which the afferent inputs come in: this is partly determined by the spatiotemporal organization within the IO (Llinás, 2009), and the isochronicity of the conduction time along the sagittal band (Fukuda et al., 2001; but see Baker and Edgley, 2006a,b; Lang et al., 2006a). The final result is ultimately that sagittal bands of CSs respond preferentially at a frequency neighboring 10 Hz. This imposed rhythmicity onto the PCs would have important implications from the standpoint of spatiotemporal time encoding in the cerebellar cortex, favoring events separated by 100 ms (Welsh, 2002), but also a capacity to affect the cerebellar cortex network memory (Ito, 1989, 2006) at that frequency. This packaging of information using oscillatory activity has been identified within hippocampal and entorhinal systems (Jensen and Lisman, 2005; Buzsaki and Moser, 2013). In the cerebellum, while multiple synapses are likely to change with repeated circuit stimulation (Gao et al., 2012), oscillation with memory has scarcely been addressed, but are likely to play a role (D’Angelo et al., 2011). There is also evidence of cerebellar fMRI activity being linked to slow-wave oscillations during sleep, which was shown to have a role in memory processes (Dang-Vu et al., 2008).

### GRANULE CELL LAYER 4–25 Hz OSCILLATIONS

While connectivity in the olivocerebellar pathway shows a direct and tightly interconnected system, the mossy fiber afferent system of the cerebellar cortex is strikingly different. Mossy fiber input interacts at multiple levels, connecting with many interneurons before reaching the final output layer, the PCs (Llinás et al., 2004; Ito, 2010). The main target of the mossy fiber pathway, the GCL, is a heavily interconnected network composed of the cell bodies of granule, Golgi, Lugaro, and unipolar brush cells. The Golgi–granule cell network, which acts to integrate the incoming signals from mossy input, is a very active and dynamic local network. Both granule and Golgi cells receive excitatory inputs from the mossy fibers. Golgi cells are inhibitory interneurons whose axonal projections mostly remain within the GCL, while granule cell axons extend up to the molecular layer where they bifurcate and synapse with the dendritic projections of the PC (Eccles, 1967; Kalinichenko and Okhotin, 2005; Ito, 2010). This connectivity is illustrated in **Figure 2**.

While the anatomy of the mossy fiber pathway is well-known (Eccles et al., 1967), the 4–25 Hz rhythmic oscillatory activity in the GCL was only recently reported. While exploring the cerebellar cortex for CS activity in the awake behaving monkey, Pellerin and Lamarre (1997) heard rhythmic “background” activity within the GCL when electrodes entered the PM of the monkey. The rhythmic activity which they heard was in reality a bursty multi-unit GCL discharge at ~14 Hz: by adapting filters on the electrophysiological signal for the observation of LFPs, rhythmic oscillations were recorded in the form of waxing and waning spindles. This rhythmic signal was recorded while the rhesus monkey remained immobile but attentive to the environment; it decreased with the initiation of movement. This rhythmic activity was also affected by the level of arousal of the animal and was modulated in amplitude by both sensory events and motor output. In each situation, oscillatory power was shown to predictably diminish with reduced levels of arousal, input of a sensory stimulus, and the initiation of motor task. A similar oscillatory phenomenon was also reported shortly afterward in the rodent cerebellar cortex, at ~7 Hz in the GCL of Crus II. These oscillations were present during immobility and were also stopped by the initiation of movement (Hartmann and Bower, 1998). Early accounts report certain slow field activity which could be related to GCL oscillations (Brookhart, 1960). As explained, this GCL rhythmic activity is tightly correlated with multi-unitary GCL activity, obvious from the LFP recordings. The oscillatory activity was found to be generally synchronous across separate recording sites in Crus II both within and across hemispheres in the GCL. These two papers marked the beginning of the identification of another rhythmic phenomenon in the cerebellar cortex, in this case rather than affecting the PCs through the CFs, the rhythms influence cerebellar output through the mossy fiber relay in the GCL.

Many similarities were evident in those two papers: (1) the LFP oscillations were clearly related to the GCL activity; (2) these oscillations were best recorded during periods of immobility of the animal; and (3) these oscillations were spindle-shaped and lasted several cycles. Thus, despite a difference in frequency band (monkey 14–20 Hz, rodent 7–8 Hz) and a minor difference in localization (monkey PM, rodent Crus II), the low-frequency rhythms were recorded optimally under similar conditions: the power of the oscillations was highest when animals were immobile, showing spindle-shaped oscillations that lasted over several cycles. Following these publications, unit activity in relation to the oscillatory LFPs in the cerebellar cortex was further defined in the primate showing: (a) the best cellular relationship being multi-unit granule cells; (b) second best being PC simple spikes; and (c) no obvious relationship between CF activation and beta-range LFPs (Courtemanche et al., 2002). One behavioral distinction here was that the animals were asked to perform several tasks, all of which essentially were related to the concept of active expectancy, or “waiting for the proper time to trigger the movement” (Courtemanche et al., 2002). In addition, with the potential that the 15–25 Hz GCL oscillations might need programmed movement to take place, we showed that GCL oscillations increase if the animal was in a state of passive expectancy, the spindles lasting as long as the waiting period (Courtemanche et al., 2002).

In the years since the Pellerin and Lamarre (1997) and the Hartmann and Bower (1998) papers, there have been several reports that have defined the rhythmic properties of the cellular components of the GCL, as well as the behavioral states which influence the development and efficiency of these rhythms. The cellular properties of the GCL components and their synaptic organization provide an ideal environment for the development and maintenance of rhythmic low-frequency activity. Granule cells possess specific slow potassium channels that enable 3–12 Hz bursting and resonance (D’Angelo et al., 2001). Golgi cells display specific firing properties that promote the rhythmic inhibition of granule cells, as demonstrated during both *in vitro* and *in vivo* recordings. Golgi cells possess intrinsic pacemaking and resonance, seen *in vitro* with the regular beating of Golgi cells at frequencies within the theta-band range (Dieudonn, 1998; Forti et al., 2006; Solinas et al., 2007). In addition, Dugué et al. (2009) showed in the rodent that Golgi cells could certainly be influenced by the oscillatory phenomenon in the GCL: by manipulating electrical synapse connectivity, they showed that Golgi cells could form a network capable of maintaining 4–25 Hz resonance in the GCL circuitry. These findings also complement *in vivo* recordings, where unitary activity shows spontaneous rhythmic firing found in both awake and anesthetized animals (Edgley and Lidierth, 1987; Vos et al., 1999; Holtzman et al., 2006). Rodent GCL theta-range oscillations, under urethane anesthesia, show similar oscillatory frequencies as in the awake animal (Robinson et al., 2009), as is the case for urethane anesthesia and the hippocampal or entorhinal theta oscillations (Glasgow and Chapman, 2007; Zhang et al., 2010).

Golgi cells have many diverse synaptic connections with both granule and other Golgi cells; one of those types consists in Golgi–Golgi electrical synapses. Gap junction proteins connexin36 (Cx36) have been identified in the GCL and molecular layers of the cerebellar cortex, located primarily on apical dendrites of Golgi cells (Condorelli et al., 2000; Ray et al., 2006; Vervaeke et al., 2010). Cx36 gap junctions have been associated with the synchronizing of inhibitory networks (Deans et al., 2001) and may be a contributing factor to network synchrony within the GCL (Vervaeke et al., 2010). Functionally, electrical coupling between Golgi cells serves to promote synchronization of their rhythmic firing as a population, thus providing synchronous inhibition to granule cells. Dugué et al. (2009) showed that Golgi cells could maintain 4–25 Hz resonance in the GCL circuitry. In addition, Vervaeke et al. (2010) also demonstrated that in paired recordings of Golgi cells in the absence of mossy fibers, Golgi cells maintain synchronous signals, pointing to the capacity of the GCL to possibly develop or maintain low-frequency rhythms.

An additional cellular component, the Lugaro cell, may also act as a temporal coordinator in the GCL by modulating and synchronizing activity (Dieudonné and Dumoulin, 2000). Lugaro cells are inhibitory interneurons connected to Golgi cells that transversely connect different sites of the GCL (Lainé and Axelrad , 1996). Lugaro cells possess a myelinated axon, permitting them to connect sites with a faster response than the parallel fibers. An interesting property of Lugaro cells is that they produce oscillatory inhibitory post-synaptic current (IPSCs) in the membrane potential of Golgi cells following the administration of serotonin (Dieudonné and Dumoulin, 2000). This connection could thus potentially coordinate Golgi–granule local circuits in a coherent fashion. The Lugaro properties would make for a second mechanism by which a spatially defined, orthogonal network [sagittal for Golgi cells axons and Lugaro dendrites (Geurts et al., 2003), coronal for Lugaro axons], could influence the 4–25 Hz oscillations in the GCL. Intrinsically, the cell properties of both the granule cells (D’Angelo et al., 2001), and Golgi cells (Forti et al., 2006) could provide the underlying strong resonance around 4–25 Hz. These properties confer specific time windows for optimal GCL processing of information (D’Angelo, 2008; D’Angelo and de Zeeuw, 2009; D’Angelo et al., 2009). Overall, Lugaro, granule, and Golgi cells have all been reported to have distinctive properties that can facilitate rhythmicity, in which Golgi cells appear to play a pivotal role. One remaining question is whether this system can intrinsically generate these oscillations or is simply a cellular environment capable of maintaining externally driven rhythms.

#### Functional roles of 4–25 Hz GCL oscillations

To explore the functional role of the cerebellar rhythms with respect to sensorimotor systems, task-related cerebellar recordings have been compared to the rhythmic activity in other brain regions. O’Connor et al. (2002) found ~7 Hz synchronized activity across the Crus II cerebellar cortex, the contralateral primary somatosensory cortex, and mystacial pad electromyography (EMG), addressing the potential interactions across extended somatosensory processing circuitry. One of their salient results is that this coherence was more pronounced when the animal was whisking weakly or not whisking at all. This finding seems to echo previous reports concerning optimal behavior for eliciting GCL LFP oscillations, namely the little or no obvious motor output; this information was now being related to a larger coherent network for whisking.

In primate recordings, Courtemanche and Lamarre (2005), also examined the link between GCL oscillations in the 10–25 Hz band range and sensorimotor processing. In the context of active expectancy, PM GCL LFP oscillations were highly synchronized with contralateral primary somatosensory cortex (SI) rhythms. This synchrony was particularly high during the waiting period before a learned lever press, when the monkey was just lightly touching the lever in anticipation of the right time to press. Synchronization between the two regions (PM–SI) was significantly higher in active expectancy than in passive expectancy or rest, hinting that the synchronization might be related to common functional processing. Primary motor cortex vs. PM GCL 10–25 Hz oscillations seemed less linked in the context of the tasks, though active expectancy also seemed to incite the greatest synchronization. Finally, an unreported 40 Hz cerebellar cortex anterior lobe oscillation also seemed to be related to primary motor cortex oscillations in the rest condition (see **Figure 1A**). More anterior lobe recordings, presumably in the GCL, would have to be performed to substantiate those oscillations further and describe their functional significance.

Courtemanche et al. (2009) reported data about the spatial organization of cerebellar cortex GCL oscillations by simultaneously recording with two electrodes in the rhesus monkey cerebellum. Anatomically and physiologically, the cerebellar cortex can be subdivided in many spatial modules (Hawkes et al., 1997; Herrup and Kuemerle, 1997; Ebner et al., 2012), and there are particular afferent patterns that will shape the inputs to the GCL. Specifically, the predominantly sagittal arrival of mossy fiber afferents (Scheibel, 1977; Heckroth and Eisenman, 1988), and the tendency of the Golgi cells to follow the sagittal axis (Sillitoe et al., 2008) could shape how local networks are constrained physiologically. These anatomical elements appear to anisotropically limit the extent of GCL rhythmicity. In Courtemanche et al. (2009), the modularity in the GCL synchronization was sought. The dual-GCL recordings showed a primarily parasagittal organization of the GCL oscillations when the animal was at rest, with strong parasagittal LFP synchrony, and much weaker coronal synchrony. However, during active expectancy, while the sagittal cross-correlation stayed, there was a strong increase of LFP synchronization in the coronal plane. Thus, there is potentially a widening of a putative sagittal module in the context of a sensorimotor task: this could be a hint of a recruitment mechanism in order to perform a task, originating in the GCL. This widening of the electrophysiological modulation of cerebellar cortical networks also appears in the context of synchronous firing in PCs (Heck et al., 2007).

Overall, these studies have shown that GCL 4–25 Hz oscillations can serve to spatiotemporally organize the communication (1) within the GCL through the organization of the cellular networks, (2) in the output from the GCL by influencing the PCs, (3) in the spatial patterns of GCL synchronization in time, as seen in the context of functional synchronization, and (4) between the cerebellum and cerebral cortex, as seen through the cerebro-cerebellar LFP synchronization during task performance. There has been modeling of the oscillatory activity in the GCL that identifies its capacity to temporally organize the flow of inputs (Maex and De Schutter, 2005; Yamazaki and Tanaka, 2005; Ito, 2010). The GCL oscillations can thus help in the investigation of information flow throughout the cerebellar cortex and other communicating units along sensorimotor system pathways. This was shown in recent data (Courtemanche et al., 2009), adding nuance to what had already been predicted, namely that GCL oscillations at 4–25 Hz should have a patchy organization (de Zeeuw et al., 2008). Further recordings of GCL units with LFP signals will provide more information on the population specificity. Nonetheless, from the LFPs, a distinct dynamic modulation appears to exist in the GCL, with a task-related adjustment of the synchronous zones ultimately leading to optimal information processing in the cerebellar cortex.

A strategy that follows rhythmic synchronization between putative sagittal GCL-Purkinje modules could also be spatiotemporally optimal. From the standpoint of the GCL, the influence from 4 to 25 Hz rhythmicity (delays of 40–250 ms) provides the GCL sites with repeated “up-phases” lasting 50% of the rhythm cycle, amounting to periods lasting between 20 and 125 ms. These up-phases represent times when local groups of GCL neurons would be more easily excitable. This window corresponds well with certain demands imposed by the relatively slow conduction velocity of the parallel fibers (0.2–0.3 m/s; Bell and Grimm, 1969; Vranesic et al., 1994). If the objective was to simultaneously relay excitation at two cerebellar cortex sites along the parallel fibers, at these conduction velocities, the length of the parallel fiber (up to 6 mm; Brand et al., 1976) would be covered in a period of 20–30 ms. The up-states for excitation provided by the rhythmicity would thus have to last longer than 20–30 ms to provide an additional rhythmic advantage. From this calculation, a rhythm with a period of more than 20 ms, or frequencies less than 50 Hz would thus favor a spatiotemporal pattern of synchronization throughout the length of the parallel fibers.

### FAST (>150 Hz) OSCILLATIONS IN THE PURKINJE CELL LAYER

Early in the study of cerebellar physiology, Adrian had recorded fast (150–250 Hz) oscillations from the ECoG (electrocorticographic) signal on the surface of the cerebellar cortex of the anesthetized cat and rabbit (Adrian, 1935; Isope et al., 2002). The presence of these oscillations was also confirmed in other species using a similar methodology (Brookhart, 1960). At the time, these oscillations were demonstrated to specifically originate from the cerebellar cortex (Dow, 1938). In a more recent series of studies, using microelectrodes to record from within cerebellar cortex of mutant mice, this type of fast activity (>150 Hz) was recorded by Cheron et al. (2008). In a mouse model of Angelman syndrome, they found prominent fast oscillations while recording LFPs, along with single unit activity (Cheron et al., 2004, 2008; Gall et al., 2005), and confirmed with precision the link of these oscillations with PC activity. In two other recent papers, the existence of these fast oscillations in normal animals has also been confirmed (de Solages et al., 2008; Middleton et al., 2008). Experiments *in vivo* showed fast (~200–250 Hz) rhythms in normal rats (de Solages et al., 2008). The presence of such oscillations has also been confirmed by experiments *in vitro* (Middleton et al., 2008). *In vivo* experiments showed that these oscillations are robust, present mainly in the PCL, and are also affected by the recurrent PC collaterals (de Solages et al., 2008).

#### Functional specificity of fast oscillations

Fast oscillations could present a different modulatory pattern onto the cerebellar cortex circuitry. Via their localization in the PCL, they can more directly influence the motor output toward the DCN. Through recurrent collaterals, they can influence local neighboring zones and fine-tune spatially the output area (de Solages et al., 2008). However, they do not appear to show a sagittal or coronal pattern of coherence. This could be related to the restricted spatial extent of this coherence (on the order of 0.5 mm).

It is not known if these oscillations are specifically affected by movement initiation. However, they are affected by the neurophysiopathological disease state, as described below. They appear to be more pronounced in the case of specific diseases, such as Angelman syndrome, and in calretinin/calbindin knockout mice (Cheron et al., 2005). These conditions appear to exacerbate the oscillations, which will be described in section “Potential Interactions of These Oscillations in the Cerebellar Cortex: Perspectives from Movement.”

### SLOW OSCILLATIONS (≤1 Hz) IN THE CEREBELLAR CORTEX

Recently, a few research groups have identified slower oscillations in the cerebellar cortex circuitry. Specifically, one type involves slow oscillations (0.05–0.2 Hz) present in the ataxic tottering (*tg*) mouse, recorded with flavoprotein immunofluorescence (Chen et al., 2009). This mouse has defective Ca_v_2.1 (P/Q-type) voltage-gated Ca^+^^2^ channel, and suffers from short bouts of dystonia/dyskinesia. These oscillations appear to be generated intrinsically, as they are not disturbed by blocking glutamate α -amino-3-hydroxy-5-methyl-4-isoxazolepropionic acid (AMPA) receptors, and affect the cerebellar cortex cells, including PCs. The slow oscillations, which seem to increase in activity, are spontaneously present in the cerebellar cortex active area during dystonic periods. The frequency increases to values of 0.15 Hz. These oscillations are coherent with the muscular activity triggered during dystonic episodes. The capacity to record such slow oscillations in normal animals does not appear to have been reported.

The McCormick group reported a slow oscillatory activity around 1 Hz in the cerebellar cortex of ketamine-anesthetized mice, driven by the neocortical oscillatory activity (Ros et al., 2009). This type of oscillation could influence cerebellar cortex coding on a slow timescale, in normal animals. This 0.5–1 Hz slow oscillation is similar to the up/down states seen in cortex and basal ganglia, which are thought to have a cortical origin (Stern et al., 1998; Steriade, 2003). The pattern of activity seems similar to slow wave sleep activity recorded from neocortical and hippocampal sites (Clement et al., 2008). This slow cerebellar oscillation was shown to affect the multi-unit activity in the cerebellar cortex. In the awake mouse, this activity decreased in amplitude and accelerated to about 1.3 Hz. Tests showed a strong dependence of this locally generated cerebellar oscillatory activity to neocortical entrainment. The effects of these slow up/down states in the cerebellar cortex was to entrain granule cells and Golgi cells, but minimally PC simple spikes. However, the neocortical up-states seem to favor the emergence of PC CSs. Both of these recent sets of results, while coming from different phenotypes, appear to show how these slower oscillations could affect the cerebellar cortex circuitry.

## POTENTIAL INTERACTIONS OF THESE OSCILLATIONS IN THE CEREBELLAR CORTEX: PERSPECTIVES FROM MOVEMENT

In this section, we will identify potential nodes of interaction for the oscillations presented in the previous sections. We will focus on certain contexts for inspecting spatiotemporal dynamics, namely how those oscillations relate to movement and motor neuropathology. There is more data available in the literature concerning the olivocerebellar and GCL oscillatory phenomena, both at frequencies within 4–25 Hz. However when appropriate, we will also cover the potential interactions of cerebellar cortex fast (>150 Hz) and slow (=1 Hz) oscillations. The identification of potential oscillatory interactions is largely unknown from the standpoint of the experimental data available. However, we attempt educated guesses in the case of two specific contexts: the immobility/movement interface, which is a standard sensorimotor context where it is possible to identify a phase transition in the circuits (Konig and Engel, 1995; Buzsaki, 2006; Courtemanche et al., 2009; Salazar et al., 2012), and also of select “neuropathological” activity in the circuits, such as during injection of harmaline, triggering symptoms of tremor (Llinás, 2009; Park et al., 2010), or in ataxic mouse models.

### CROSSROADS AND POTENTIAL INTERACTIONS

By examining the anatomical intersections across the cerebellar circuitry, we have identified three potential interaction sites. These interaction sites constitute neuronal groups where the influence of more than one oscillatory phenomenon converges. We identified potential interactions at the following sites: at the level (1) of PCs; (2) of the DCN; and (3) at the GCL. Those sites and their connectivity are identified in the network diagram of **Figure 2**. Quite probably only for practical reasons in experimentation, many of the recordings of oscillations occurred in the posterior lobe, in the Crus II and PMs (see **Figure 2A**).

First we will describe some of the connectivity that could support these interactions. *(1) Level of the PCs. *One of the first potential sites of oscillatory interactions is at the level of the PCL (see “1” in **Figure 2B**). As a site, PCs receive, amongst other afferents, the CFs from the IO, the parallel fibers and the ascending axons from granule cells (Gundappa-Sulur et al., 1999; Bower, 2002; Ito, 2010). It is thus an area where the olivocerebellar oscillations and the GCL oscillations can converge, at similar frequencies. It is also a site where the 4–25 Hz oscillations can interact with the slow (<1 Hz) and fast (>150 Hz) oscillations. Specifically for the theta and beta bands, the way that oscillatory interactions would happen is via the convergence of the simple spike activity (influenced by the GCL oscillations – see Courtemanche et al., 2002), and the CS activity produced by the IO. *(2) Level of the DCN.* Another potential site of interaction are the cerebellar nuclei (see “2” in **Figure 2B**). The DCN receive connections mainly from PCs, but also receive collaterals from the IO and from mossy fibers (Llinás et al., 2004; Ito, 2010). The nuclei could thus be a site of interaction between the olivocerebellar and GCL oscillations. *(3) At the level of the GCL.* Along with local resonance mechanisms, another potential multi-oscillation site would be the GCL (see “3” in **Figure 2B**). The IO also send CF collaterals to the GCL (Geurts et al., 2003). Although less is known about these connections, there could be an interaction between the olivocerebellar and the GCL oscillations at this level.

### FROM IMMOBILITY TO MOVEMENT

As was discussed previously, the GCL oscillations and the IO rhythmicity do not require movement in order to occur, as they can appear spontaneously during immobility or under anesthesia. However, when there is a switch from immobility to movement, multiple experimental results point to the interruption of the GCL oscillations (Pellerin and Lamarre, 1997; Hartmann and Bower, 1998; Courtemanche et al., 2002, 2009; Courtemanche and Lamarre, 2005). Looking at what happens at interaction site #1 (**Figure 2B**), this movement initiation (or the concomitant surge in sensory input) appears to limit the capacity of PCs to follow the oscillatory influence from the GCL. From a large set of studies, it has been established that simple spikes exhibit a variety of modulation patterns relative to movement, such as movement onset-timed increases or decreases in firing rate (Lamarre and Chapman, 1986; Medina and Lisberger, 2008; Ebner et al., 2011). At or just preceding movement onset, there is an important task-related change of state in the local neuronal network. This would modify how the oscillatory activity from the IO or the GCL could maintain their influence on the cerebellar cortex local circuits. In the case of an imminent movement, one could liken the interaction between the phasic sensorimotor information processing and the oscillatory processes to a neuronal tug-of-war, where these processes compete to influence the neuronal cerebellar cortex excitability. Indeed, the neuronal populations of PCs change state when going from immobility to movement, evidenced clearly in the case of the simple spikes. Other state-related changes, such as the bi-stability capacity of PCs, which differentiate neuronal responsivity (Loewenstein et al., 2005; Schonewille et al., 2006; Shin et al., 2007), could also affect the underlying measure with which baseline oscillations can exert influence on the PC neural sheet. As for the olivocerebellar CS activity, their rate is often increased after movement initiation, showing a change of state (Kitazawa et al., 1998; Medina and Lisberger, 2008). Again, this state change is likely an attractor that will affect and deter the PCs from following GCL oscillatory activity if the involved movement is phasic. In short, it appears that movement stops GCL oscillations, decreasing their oscillatory influence on PC simple spikes. At the same time, it appears that the phasic CS activity related to movement can monopolize olivocerebellar signaling. While the picture of olivocerebellar activity inferred by CSs is not easy to identify due to their low firing rate ~1 Hz, IO activity is directly related to movement initiation (Lang et al., 2006b).

In the context of going from immobility to movement, one could identify oscillatory interactions on the basis of sensorimotor spatiotemporal influences at the level of PCs (site #1). For example, the GCL oscillations, favoring a sagittal plane organization during immobility, expand their synchronization zone in a medio-lateral fashion, for a few millimeters, presumably to better synchronize the functional aspects of involved cerebellar zones (Courtemanche et al., 2009). In a similar manner, olivocerebellar CSs are better synchronized in the sagittal plane during immobility (Lang et al., 1999; Bosman et al., 2010), and re-organize this synchrony relative to movement (Welsh et al., 1995). This new organization specific for the task at hand produces a modified population code for the PCs, which were previously under the influence of both olivocerebellar and GCL oscillations. The olivocerebellar movement mosaic also appears to obey specific neural coding parameters, bringing together networks of PCs and resetting oscillations (Leznik et al., 2002), which would strongly signal movement initiation. One would expect that for specific GCL population codes going further up to PCs (Hartmann and Bower, 2001; Lu et al., 2005), the need to group together PCs pertaining to multiple receptive fields would require a networking mechanism such as oscillations, for the collection of information to produce an imminent movement. This is comparable to identifying a mechanism to bring together the multiple components of a cerebellar map (Apps and Hawkes, 2009), as is the case in other brain networks (Moser et al., 2010). However, when movement happens, it appears that the influence from the IO and GCL oscillations decreases its stronghold on the neuronal population, to make way for the phasic coding, which possibly acts as a reset. In a stimulus–response sensorimotor context, after the stimulus is given, the oscillations could then serve a network preparation role to optimize the neural populations that will serve to produce the upcoming response. For the case of phasic sensory activity, it appears that whisker sensory input favors the synchrony of simple spikes along the transverse plane, and that synchrony of CSs favor the sagittal plane (Bosman et al., 2010). Simple spikes seem to align better on-beam during movement, i.e., in the transverse plane and following the orientation of the parallel fibers (Heck et al., 2007). These quick and apparently information-specific changes of state in the networks would then favor more task-related information processing until the oscillatory stronghold on the networks resumes, similarly to the massive movement effect seen in the LFP synchrony (Courtemanche et al., 2009).

Finally, an interaction with faster and slower oscillations can be speculated with regards to movement. We already identified that fast (>150 Hz) oscillations are present under anesthesia, suggesting they do not require movement to be present. It is not known right now if in normal circuits, fast oscillations directly influence PCs during movement. However, as seen in the pathological circuits of knockout mice, they appear to be stopped by direct tactile stimulation (Cheron et al., 2005), in a manner similar to the slower oscillations in the GCL, or IO activity. Spatially, the faster oscillations seem to group together close-by PCs within a region less than 0.5 mm (de Solages et al., 2008), and maybe to a greater spatial extent in pathological models (Cheron et al., 2005). This spatial specificity for the higher frequency oscillations in the context of the cellular entrainment points to a capacity to have more localized change leading to a more information-specific involvement. This issue would have to be looked into further.

Evaluating the capacity of the DCN (site #, **Figure 2B**) to entertain movement-related oscillatory interactions is a complex situation. Keating and Thach (1997) did not find strong evidence of rhythmicity in DCN unit firing. In a more recent report (Baumel and Cohen, 2012), there appears to be some rhythmic 7 Hz activity that can be recorded in the DCN; however, whether its source is from GCL or olivocerebellar activity cannot be determined yet. It also appears as though DCN activity could be related to rhythmicity in the electromyogram (Aumann and Fetz, 2004), playing a role in the way downward connections are affected by rhythmic efferent activity. There is a definitive advantage, though, for PC simple spikes to synchronize their activity onto common DCN target units, to increase the effectiveness of the connection (Person and Raman, 2012a,b). In this case, afferent oscillations could serve to provide the background for synchronous activity, increasing the likelihood of influencing DCN neurons via the synchronization of the PC firing. It is also known that the olivocerebellar activity can effectively influence the DCN neurons at a magnitude similar to the influence of simple spikes (Lang and Blenkinsop, 2011). This influence is beginning to be explored. It is clear, from recordings in IO units, that their rhythmic subthreshold oscillations, should they compound together, can provide the capacity to transmit rhythmic CSs to efferent targets (Chorev et al., 2007). Under the influence of harmaline, DCN neurons can be driven to fire in synchrony with the olivary activity (De Montigny and Lamarre, 1973; Lamarre, 1994). As for the olivocerebellar interactions affecting the GCL (site #3), in the same way that the IO can transmit rhythmic spikes to the PCs or to the DCN (Chorev et al., 2007), there is anatomical evidence that they can also influence the GCL, but a specific physiological relationship has not been reported or systematically studied.

### NEUROPATHOLOGICAL ASPECTS

The link with oscillations and neuropathology for the cerebellar cortex is not as clear as is the case for the basal ganglia (Hutchison et al., 2004; Gatev et al., 2006). In Parkinsonian models, dopamine depletion leads to an increase in the oscillatory phenomena (Bergman et al., 1998; Hammond et al., 2007; Lemaire et al., 2012). Not all of the oscillations described in the above sections are primarily present in neuropathophysiological models; on the other hand, certain neuropathophysiological models appear to have enhanced types of oscillations. One interesting case, resembling essential tremor (Deuschl and Elble, 2000), is with the hyperrhythmicity in the olivocerebellar pathway produced by the administration of systemic harmaline to the animal, or directly in the IO. In these conditions, a strong IO population synchrony effect is produced by harmaline. The IO hypersynchrony increases the capacity to emit CSs and brings together much larger populations of PCs (see site #1, **Figure 2B**; Sugihara et al., 1995). The effect of this hypersynchrony on the GCL oscillations (relative to site #2) is unknown. Such a hypersynchronous population pattern of activity could transmit rhythmic signals to the GCL, and drive the networks of the GCL (site #3) in a non-specific way (for example, triggering heightened diffuse synchrony in a manner out of the usual parasagittal plane dominance). This would have to be tested. Finally, with respect to oscillations in the cerebellar cortex at 4–25 Hz, Cheron et al. (2009) identified a role for BK (big potassium) calcium-activated potassium channels in PCs and Golgi cells. In mice where this channel has been knocked out, and consequently rendered ataxic, PC simple spikes show strong rhythmicity in the 15 Hz range. When comparing the LFPs with cell activity, the strong 15 Hz LFP component was tightly related to unit firing: PC simple spikes and CSs, and Golgi cells were phase-locked with the LFP. This model provides the opportunity to study multiple oscillatory interactions, both at the level of the PCL or the GCL. Another component is that the LFP oscillation synchrony appears to be less aligned with the sagittal plane than would be expected: the synchronization appears broad and strong in *both* transverse and sagittal orientations. This component would have implications on how the cerebellar cortex networks organize themselves relative to the sensorimotor maps, specifically by affecting DCN elements in a hypersynchronous mode, potentially removing the muscle/movement selectivity typically seen in ataxia.

Another disease which is related to the 4–25 Hz frequency range is the case of essential tremor, a disorder which is primarily characterized by a 4–12 Hz tremor (Lamarre, 1994; Pinto et al., 2003). A well-known clinical model of this disorder is the previously mentioned harmaline model. Harmaline-induced tremor is characterized by a strong, near 10 Hz tremor of the animal, very similar to the 4–12 Hz tremor observed in essential tremor patients (Lamarre, 1994). The mechanism of action of harmaline is thought to be a potentiation of Ca_v_3.1 T-type Ca^2+^ channels in the IO that leads to strong subthreshold oscillations of IO cells and increase the probability of CF action potentials. Due to the strong CF–PC synapses, PCs are entrained to fire CSs at ~10 Hz. This rhythmic activity, starting at the IO, spills over and then entrains the synchrony of upstream nodes of the Guillain–Mollaret triangle (rubral nucleus, olivary nucleus, and cerebellum), manifesting as tremor. This same basic mechanism is thought to be the cause of essential tremor, with a pathologically oscillating network comprising the IO, the cerebellum, the thalamus, and the motor cortex (Raethjen and Deuschl, 2012). Interestingly, deep brain stimulation of the ventral intermediate nucleus of the thalamus (Vim) is an efficacious treatment for essential tremor patients (Lozano and Lipsman, 2013). Although not monosynaptically connected to the IO, the Vim primarily receives input from the cerebellar nuclei, which receive input both directly and indirectly (through PCs) from the IO. With regard to the pathogenesis of essential tremor, the thalamus is considered to play an important role in coupling different regions of the nervous system (Buzsaki and Draguhn, 2004), with the olivocerebellar and primary motor cortex connections being of particular interest in essential tremor.

Schnitzler et al. (2009) found oscillatory coupling at tremor frequencies between brain areas, including subcortical areas such as the thalamus and cerebellum. Using magnetoencephalography (MEG), they showed cerebro-muscular and cerebro-cerebral coupling during a motor task. Additionally, Hanson et al. (2012) identified ensembles of Vim neurons that were oscillating at near-tremor frequencies between 2.5 and 7.5 Hz in essential tremor patients. However, there was no clear phase relationship between these oscillating units and tremor. Furthermore, Popa et al. (2013) found that repetitive bilateral transcranial magnetic stimulation of the posterior cerebellum of essential tremor patients improved all symptoms (e.g., tremor reduction, writing, pouring). The effects were progressive (ramping up over time), and persisted for up to 3 weeks after treatment (Popa et al., 2013). An additional benefit was that the functional connectivity of the cerebello-thalamo-cortical (CTC) network, evaluated using fMRI, was improved. When compared to controls, essential tremor patients had less functional connectivity within the CTC at baseline, but did show a partially re-established network connectivity of the CTC following the fifth day of treatment (Popa et al., 2013). With its neural and behavioral effects, this treatment seems promising. Although the pathophysiology of essential tremor remains elusive, the consensus remains that its genesis is related to a pathological synchrony of multiple areas, namely the olivocerebellum, thalamus, and motor cortex (Deuschl and Bergman, 2002).

Two other examples can be used to illustrate a neuropathological pattern at slower and faster frequencies. In the tottering mouse, the oscillations in the fluorescence measures (<1 Hz) seem to affect a large component of the cerebellar cortex circuits, including PCs (Chen et al., 2009). In this case, speculating on interactions of the oscillations at those nodes, a potential effect is that the PC output will again affect the DCN in a hypersynchronous fashion. This effect seems to be even more important during dystonic episodes, enough to trigger related rhythmic muscle contractions. Finally, in experiments on cerebellar mutant mice from the Chéron laboratory, faster oscillations seem to show heightened spatiotemporal synchrony. This is the case of the Angelman mouse model, where it appears like the fast (>150 Hz) oscillations in the cerebellar cortex are hypersynchronous for zones up to 1 mm (Cheron et al., 2005), a zone larger than normal fast coherence zones. Some of those fast oscillations are also seen in calretinin/calbindin mutant mice (Cheron et al., 2004, 2008), affecting the PC layer. In this model, the synchronization of fast oscillations appeared to follow the coronal plane, in line with the parallel fiber orientation, for a range up to 2 mm. For both these models, neural activity appears to show an increased synchrony at the level of the cerebellar cortex. This would also lead to a pattern of activity going to the DCN that lacks spatiotemporal selectivity.

In determining the effects of network oscillations in the cerebellar cortex, it appears that there are many different oscillatory phenomena that can coexist. At the same time, the potential for their interactions warrants that we define where and how they would influence one another, at the level of specific cells. We focused here on the PCL, the DCN, and the GCL. Finally, those interactions can be circumscribed in terms of certain behavioral conditions or circuit pathology. Future exciting research will firm up certain elements, but presently it appears that the immobility/movement interface is potentially influenced by the slow (<1 Hz), theta/beta rage (4–25 Hz), and fast (>150 Hz) oscillations. Certain rodent models also permit the evaluation of a greater range of interactions and effects on movement, where hypersynchronous rhythmicity can adversely affect movement control.

## GOING OUT OF THE CEREBELLUM, AND CONCLUSION

There is mounting evidence that cerebellar oscillations can interact with cerebral oscillations, potentially providing a long-range synchronization mechanism. These interactions have been identified in the rodent, the primate, and humans (O’Connor et al., 2002; Courtemanche and Lamarre, 2005; Soteropoulos and Baker, 2006; Kujala et al., 2007). More recent techniques for implantation of multiple microelectrodes over long periods of time are likely going to inform us about the role of the oscillations at various frequencies in triggering and modulating functional patterns of coherence in cerebro-cerebellar networks. Namely, in the context of this review, an important component is the temporal aspect of the flow of activity through and inside the cerebellum. What could be the potential roles that temporally patterned activity from the cerebellum would bring?

A first point of view is functional. A functional temporal aspect, focusing on oscillations, necessarily will rely on the structural aspects of the putative oscillators, basing interactions on the spatiotemporal properties of the neural activity. From many points of view, the cerebellum should provide accurate computations about the state of the world around us, and provide us with an enhanced capacity to further influence our environment by predicting our, and its, future state (Paulin, 1993; Bell et al., 1997, 2008; Courchesne and Allen, 1997). Oscillations in cerebellar circuits can certainly contribute to this time-dependent process, and help relate the cerebellar activity to other structures of the sensorimotor systems. Such rhythmicity could serve to synchronize its internal activity in a dynamic networks perspective but also ultimately to synchronize the activity of distant brain areas (Schnitzler and Gross, 2005). As such, with its long-range afferent input and long-range efferent penetration, the cerebellum, itself using oscillations and synchrony to coordinate its own components, could also act as a large-scale network synchronizer, via its synchronizing influences and buffering delay lines. This is akin to a role in helping to time neural operations in other structures (Llinás, 2011).

A second point of view when illustrating cerebro-cerebellar oscillatory interactions is more mechanistic. Cerebellar oscillations would influence the spatial and temporal patterns of activity in the cerebellar circuits, and the communications with the cerebrum. Cerebellar oscillations could also enhance communication with outside structures at precise times. One method to temporally control the flow of activity in a given structure is through oscillatory modulation of short periods of activity, separated by equally short silences (Sejnowski and Paulsen, 2006). Such activity has better temporal predictability. In the case of sensorimotor behavior, pre-movement oscillations in “motor” cerebral cortical areas have been identified for some time (Murthy and Fetz, 1992; Sanes and Donoghue, 1993). This corroborated, at the level of local recordings with electrodes having the capacity to resolve cells, certain elements that had been identified in earlier studies focusing on electroencephalographic (EEG) or ECoG signal (e.g., Bouyer et al., 1981; Pfurtscheller, 1981). The study of a more global sort of brain activity, from EEG to LFPs, and more recently MEG, brought a different perspective to researchers looking for cortical coding mechanisms of movement. These studies thus brought a new spin to traditional stories of information processing in sensorimotor circuits, adding a potential for oscillatory activity helping in the temporal control of the formation of neuronal circuits, a story already in full force for years in other brain areas such as the hippocampus (reviewed in Buzsaki and Draguhn, 2004; Buzsaki, 2006). The formation of systemic and local networks through their temporal properties is certainly an important component of the definition of task-related populations (Schnitzler and Gross, 2005). Such sensorimotor coding based on timed networks has been shown for cerebral mechanisms (Roelfsema et al., 1997; Singer et al., 1997; Buzsaki and Draguhn, 2004; Salazar et al., 2012), but temporal coding through oscillatory networks could even progress downward, affecting cerebral-to-spinal communications in LFP and EMG beta-range components (Baker et al., 1997), It has already been seen in MEG signals with gamma synchronization serving to organize corticospinal relationships (Schoffelen et al., 2005).

If oscillations play an important role in cerebellar circuitry, these rhythmicities need to serve to define co-active neural populations and to shape the modes of communication between those populations. While the anatomical connectivity must initially determine the way by which populations are defined, having been well studied by numerous researchers for cerebellar connectivity (Voogd, 1992; Parent, 1996; Voogd and Glickstein, 1998; Voogd and Paxinos, 2004) and cerebro-cerebellar relationships (Bloedel and Courville, 1981; Brodal et al., 1997; Schmahmann and Pandya, 1997; Middleton and Strick, 2000; Strick et al., 2009), the cerebellar circuits must also be defined spatiotemporally by the flow of neural activity at given points in time through the networks. Certain principles are often guides here: (1) the size of the interacting population is usually inversely proportional to the frequency of the oscillations, so the higher the frequency, the spatially smaller the involved circuits, while slower oscillations tend to integrate larger circuits through the loop delays (Buzsaki and Draguhn, 2004); and (2) networks with similar frequencies can more readily synchronize in a cooperative manner (Strogatz and Stewart, 1993; Strogatz, 2003).

Oscillations at slower frequencies thus appear to have a capacity to link together larger networks, or more distant components of larger networks. From this standpoint, oscillations at <1 Hz are likely to bring together the largest networks, as shown by Ros et al. (2009). However, a close second are the theta/beta-range oscillations (4–25 Hz), which appear to be coherent with cerebral cortex activity (O’Connor et al., 2002; Courtemanche and Lamarre, 2005). Finally faster oscillations (>150 Hz) appear less likely to have a cerebro-cerebellar role, potentially influencing more local circuit patterning. How oscillations help form a coherent network might also be as important as the oscillations’ role in segmenting specific networks, both contributing to dynamic routing (Moser et al., 2010). Both of these effects could be beneficial for sensorimotor operations; the former could potentially unite cerebral and cerebellar populations into a coherent representation, the latter could potentially distinguish between different subpopulations of the cerebellum and cerebrum for a more precise definition of a task-related (or operation-related) network.

## Conflict of Interest Statement

The authors declare that the research was conducted in the absence of any commercial or financial relationships that could be construed as a potential conflict of interest.
